# Diabetes Is the Strongest Predictor of Limited Exercise Capacity in Chronic Heart Failure and Preserved Ejection Fraction (HFpEF)

**DOI:** 10.3389/fcvm.2022.883615

**Published:** 2022-05-26

**Authors:** Venera Berisha-Muharremi, Michael Y. Henein, Frank L. Dini, Edmond Haliti, Ibadete Bytyçi, Pranvera Ibrahimi, Afrim Poniku, Arlind Batalli, Rina Tafarshiku, Shpend Elezi, Gani Bajraktari

**Affiliations:** ^1^Medical Faculty, University of Prishtina, Prishtina, Kosovo; ^2^Clinic of Endocrinology, University Clinical Centre of Kosova, Prishtina, Kosovo; ^3^Department of Public Health and Clinical Medicine, Umeå University, Umeå, Sweden; ^4^Cardiovascular Diseases Unit 1, Cardiac, Thoracic and Vascular Department, University of Pisa, Pisa, Italy; ^5^Clinic of Cardiology, University Clinical Centre of Kosova, Prishtina, Kosovo

**Keywords:** diabetes mellitus, heart failure, 6-min walk test, exercise capacity, Doppler echocardiography

## Abstract

**Background and Aim:**

Type 2 diabetes mellitus (T2DM) is a known risk factor in patients with heart failure (HF), but its impact on phenotypic presentations remains unclear. This study aimed to prospectively examine the relationship between T2DM and functional exercise capacity, assessed by the 6-min walk test (6-MWT) in chronic HF.

**Methods:**

We studied 344 chronic patients with HF (mean age 61 ± 10 years, 54% female) in whom clinical, biochemical, and anthropometric data were available and all patients underwent an echo-Doppler study and a 6-MWT on the same day. The 6-MWT distance divided the cohort into; Group I: those who managed ≤ 300 m and Group II: those who managed >300 m. Additionally, left ventricular (LV) ejection fraction (EF), estimated using the modified Simpson's method, classified patients into HF with preserved EF (HFpEF) and HF with reduced EF (HFrEF).

**Results:**

The results showed that 111/344 (32%) patients had T2DM, who had a higher prevalence of arterial hypertension (*p* = 0.004), higher waist/hips ratio (*p* = 0.041), higher creatinine (*p* = 0.008) and urea (*p* = 0.003), lower hemoglobin (*p* = 0.001), and they achieved shorter 6-MWT distance (*p* < 0.001) compared with those with no T2DM. Patients with limited exercise (<300 m) had higher prevalence of T2DM (*p* < 0.001), arterial hypertension (*p* = 0.004), and atrial fibrillation (*p* = 0.001), higher waist/hips ratio (*p* = 0.041), higher glucose level (*p* < 0.001), lower hemoglobin (*p* < 0.001), larger left atrium (LA) (*p* = 0.002), lower lateral mitral annular plane systolic excursion (MAPSE) (*p* = 0.032), septal MAPSE (*p* < 0.001), and tricuspid annular plane systolic excursion (TAPSE) (*p* < 0.001), compared with those performing >300 m. In the cohort as a whole, multivariate analysis, T2DM (*p* < 0.001), low hemoglobin (*p* = 0.008), atrial fibrillation (*p* = 0.014), and reduced septal MAPSE (*p* = 0.021) independently predicted the limited 6-MWT distance.

In patients with HFpEF, diabetes [6.083 (2.613–14.160), *p* < 0.001], atrial fibrillation [6.092 (1.769–20.979), *p* = *0.00*2], and septal MAPSE [0.063 (0.027–0.184), *p* = *0.002*], independently predicted the reduced 6-MWT, whereas hemoglobin [0.786 (0.624–0.998), *p* = 0.049] and TAPSE [0.462 (0.214–0.988), *p* = 0.041] predicted it in patients with HFrEF.

**Conclusion:**

Predictors of exercise intolerance in patients with chronic HF differ according to LV systolic function, demonstrated as EF. T2DM seems the most powerful predictor of limited exercise capacity in patients with HFpEF.

## Introduction

Heart failure (HF) has become a major public health problem in the past decades (1, 2), and it remains a clinical syndrome with poor prognosis in both patients with reduced ejection fraction (HFrEF) and preserved (HFpEF) (3–6). In those patients, exercise intolerance is one of the most important clinical manifestations and has been shown to be a strong predictor of all-cause mortality (7). Assessment of exercise capacity using the 6-min walk test (6-MWT) has been used as a simple, reproducible, and inexpensive method (8, 9). Indeed, 6-MWT has been shown to have a good correlation with objective measures of exercise tolerance, such as exercise duration and oxygen uptake at peak exercise (10). Type 2 diabetes mellitus (T2DM) is one of the most frequently seen risk factors and comorbidities in patients with congestive heart failure (CHF) (11, 12), and it adversely affects outcomes in these patients (13, 14). The impact of T2DM on different phenotypic presentations of HF, especially in patients with HF and preserved ejection fraction (HFpEF), remains unclear (15, 16). Impaired energy metabolism and muscle fiber-type switches (17, 18) found in T2DM, similar to what is seen in CHF, have been previously shown. Accordingly, it can be assumed that T2DM may further reduce the aerobic capacity of patients with HF as a potential mechanism for the known limited exercise tolerance, as has been previously suggested (16, 19, 20). However, the evidence regarding the direct relationship between 6-MWT and phenotypic type of HF, reduced EF (HFrEF), and preserved (HFpEF), remains lacking. In this study, we aimed to investigate the direct impact of T2DM on the reduced 6-MWT distance in patients with CHF due to various presentations, HFrEF and HFpEF.

## Methods

### Study Population

We studied 344 (mean age 61 ± 10 years, 54% female) patients with the clinical diagnosis of symptomatic CHF, and New York Heart Association (NYHA) functional class I–III, secondary to ischemic or non-ischemic etiology, based on the current definitions (21). Patients were referred to the Clinic of Cardiology, University Clinical Centre of Kosovo, between May 2013 and September 2017. At the time of the study, all patients were on optimum HF medications, optimized at least 2 weeks prior to enrollment. Based on patient's symptoms and renal function: 85% were receiving ACE inhibitors or ARB, 76% beta-blockers, 11% calcium-blockers, 8% digoxin, 54% spironolactone, and 58% diuretics. Of the enrolled patients, 45% had ischemic etiology, 38% hypertensive, and 17% had unknown etiology. Furthermore, 17% of the included patients were in atrial fibrillation. Patients with clinical evidence for cardiac decompensation (NYHA class IV, those with peripheral edema), limited physical activity due to factors other than cardiac symptoms (e.g., arthritis), severe mitral regurgitation, more than mild renal failure (in patients with raised creatinine, the glomerular filtration rate (GFR) was measured and patients with values <60 ml/min/1.73 m^2^ were excluded), chronic obstructive pulmonary disease or those with recent acute coronary syndrome, stroke, or anemia were excluded from the study. Type 2 DM was defined as a fasting blood glucose level ≥7.0 mmol/L, a glycohemoglobin A1c (HbA1c) level ≥6.5%, and/or the need for oral hypoglycemic medications or insulin. All patients gave written informed consent to participate in the study, which was approved by the local Ethics Committee.

### Data Collection

Detailed history and clinical assessment were obtained in all patients, in whom routine biochemical tests were also performed, such as hemoglobin, lipid profile, blood glucose level, and kidney function tests. Estimated body mass index (BMI) was calculated from weight and height measurements. Body surface area (BSA) was calculated using the Du Bois formula: BSA (m^2^) = 0.007184 × (height in cm) ^0.725^ × (weight in kg)^0.425^ (22). Waist and hip measurements were also made and a waist/hips ratio was calculated.

### Echocardiographic Examination

A single operator performed all echocardiographic examinations using a Philips Intelligent E-33 system with a multi-frequency transducer, and harmonic imaging as appropriate. Images were obtained with the patient in the left lateral decubitus position and during quiet expiration. Measurements of interventricular septal thickness, posterior wall thickness, and LV dimensions were made at end-diastole and end-systole, as recommended by the American Society of Echocardiography (23).

Left ventricular volumes and EF were calculated from the apical 2 and 4 chamber views using the modified Simpson's method. Ventricular long axis motion was studied by placing the M-mode cursor at the lateral and septal angles of the mitral annulus and the lateral angle of the tricuspid annulus. The total amplitude of ventricular long-axis motion was measured as previously described (24) from peak inward to peak outward points. The indices were registered as lateral and septal mitral annular plane systolic excursion (MAPSE) and tricuspid annular plane systolic excursion (TAPSE). LV and right ventricular (RV) long-axis myocardial velocities were also studied using the Doppler myocardial imaging technique. From the apical 4-chamber view, longitudinal velocities were recorded with the sample volume placed at the basal part of LV lateral and septal segments as well as the RV free wall. Systolic (s′) as well as early and late (e′ and a′) diastolic myocardial velocities were measured with the gain optimally adjusted. A mean value of lateral and septal LV velocities was calculated. The left atrial diameter was measured from aortic root recordings with the M-mode cursor positioned at the level of the aortic valve leaflets.

Diastolic LV and RV functions were assessed from filling velocities using spectral pulsed wave Doppler with the sample volume positioned at the tips of the mitral and tricuspid valve leaflets, respectively, during a brief apnea. Peak LV and RV early (E wave) and late (A wave) diastolic velocities were measured and E/A ratios were calculated. E wave deceleration time (DT) was also measured from the peak E wave to the end of its deceleration. The E/e′ ratio was calculated from the trans-mitral E wave and mean lateral and septal segments myocardial e′ wave velocities. The LV filling pattern was considered “restrictive” when the E/A ratio was >2.0, the E wave deceleration time <140 ms, and the left atrium (LA) dilated >40 mm in transverse diameter (25). Total LV filling time was measured from the onset of the E wave to the end of the A wave and ejection time from the onset to the end of the aortic Doppler flow velocity.

Mitral regurgitation severity was assessed by color and continuous wave Doppler and was graded as mild, moderate, or severe according to the relative jet area to that of the LA as well as the flow velocity profile, in line with the recommendations of the American and European Society of Echocardiography (26, 27). Similarly, tricuspid regurgitation was assessed by color Doppler and continuous-wave Doppler. Retrograde trans-tricuspid pressure drop >35 mmHg was taken as evidence for pulmonary hypertension (27, 28). All M-mode and Doppler recordings were made at a fast speed of 100 mm/s with a superimposed ECG (lead II).

### 6-Min Walk Test

Within 24 h of the echocardiographic examination, a 6-MWT was performed on a level hallway surface, administered by a specialized nurse who was blinded to the results of the echocardiogram. According to the method of Gyatt et al. (29), patients were informed of the purpose and protocol of the 6 MWT, which was conducted in a standardized fashion while patients were on their regular medications (30, 31). A 15-m flat, obstacle-free corridor was used and patients were instructed to walk as far as they can, turning 180 degrees after they have reached the end of the corridor, during the allocated time of 6 min. Patients walked unaccompanied so as not to influence their walking speed. At the end of the 6 min, the supervising nurse measured the total distance walked by the patient.

### Statistical Analysis

Data are presented as mean ± SD or proportions (% of patients). Continuous data were compared with two-tailed unpaired Student's *t*-test and discrete data with a chi-square test. Correlations were tested with Pearson's coefficients. Predictors of the 6MWT distance were identified with univariate analysis, and multivariate logistic regression was performed using the step-wise method. A significant difference was defined as *p* < 0.05 (two-tailed). Patients were divided according to their ability to walk >300 m into good and limited exercise performance groups, and were compared using unpaired Student's *t*-test. Additionally, patients with HFpEF (LVEF ≥ 40%) were compared with those with HFrEF (LVEF < 40%) using the unpaired *t*-test. Due to the possible interaction of age with echocardiographic parameters, we used the general linear model to compare age-adjusted mean values of echocardiographic indices between groups.

## Results

### Patients With HF and T2DM vs. Patients With HF but No T2DM

**Figure 1:**
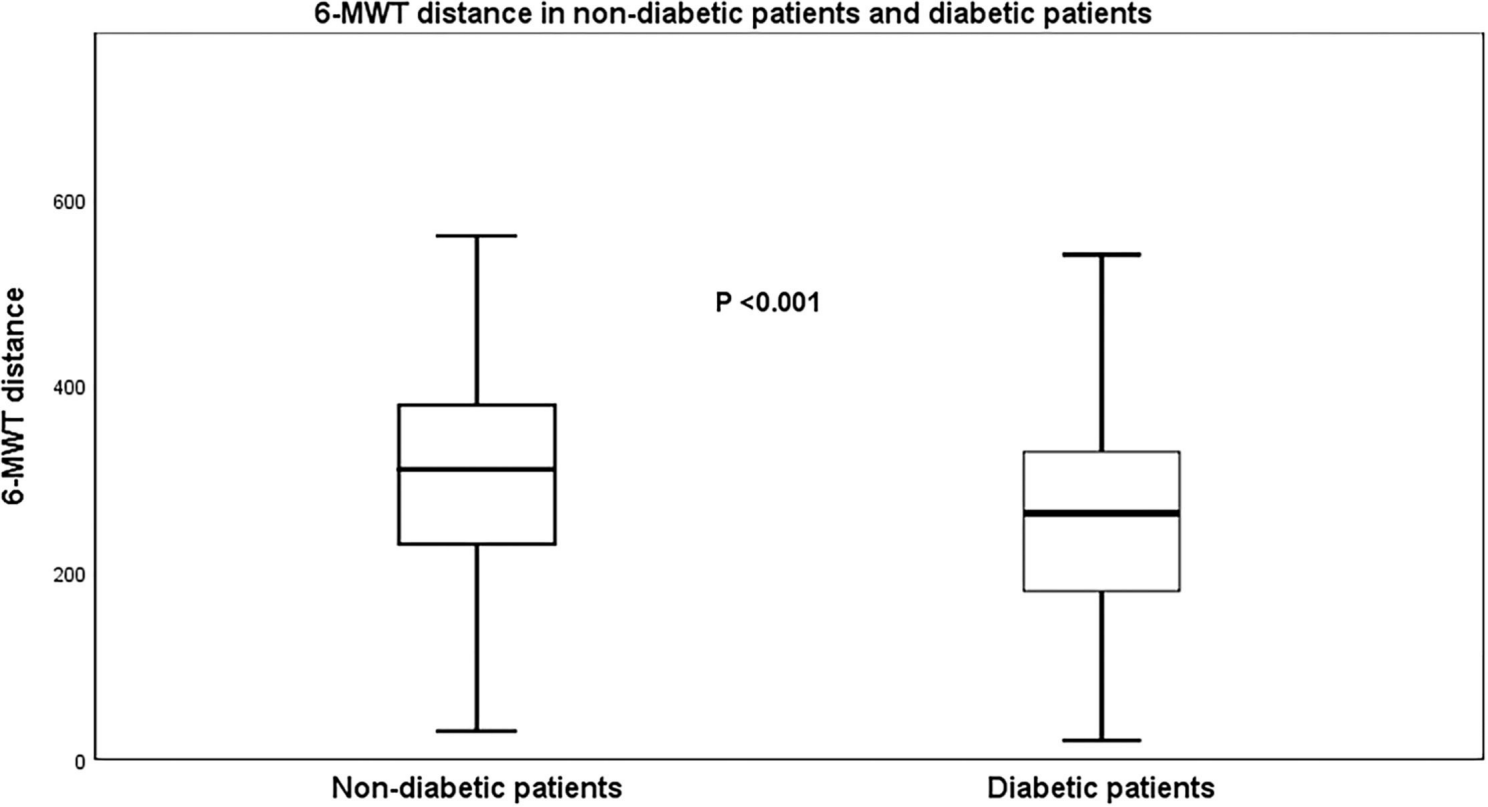
A 6-min walk test (6-MWT) distance in non-diabetic and diabetic patients with heart failure (HF).

Patients with HF and T2DM had a higher prevalence of arterial hypertension (*p* = 0.004), higher waist/hips ratio (*p* = 0.041), higher creatinine (*p* = 0.008), urea (*p* = 0.003), and lower hemoglobin (*p* = 0.001), and completed the 6-MWT for a shorter distance (<0.001) than those with HF but no T2DM (Table 1; Figure 1). The rest of the clinical indices and echocardiographic parameters were not different between groups (Table 2).

**Table 1:** Clinical data in diabetic and non-diabetic patients with chronic heart failure (HF).

**Variable**	**Non-diabetic**	**Diabetic**	***p*** **value**
	**(*n* = 233)**	**(*n* = 111)**	
Age (years)	61 ±9	62 ±8	0.213
Female (%)	55	53	0.817
Smoking (%)	28	24	0.517
Arterial hypertension (%)	78	62	0.004
Waist/hips ratio	0.95 ±0.05	0.97, 0.05	0.041
Body-mass index (kg/m^2^)	28.2 ±4.2	29.1, 5	0.094
Body-surface area (m^2^)	1.88 ±1.6	1.89, 1.7	0.551
Fasting glucose (mmol/L)	5.7 ±1.3	9.4, 3.6	<0.001
Total cholesterol (mmol/L)	4.7 ±1.2	4.6, 1.3	0.805
Triglycerides (mmol/L)	1.6 ±0.8	1.8, 0.9	0.129
Blood urea nitrogen(mmol/l)	8.3 ±4.2	10, 4.8	0.003
Creatinine (μmol/L)	95 ±34	108, 51	0.008
Hemoglobin (g/dl)	12.7 ±1.6	12.0, 2.0	0.001
6-minute walk distance (m)	307 ±111	258, 109	<0.001
Baseline heart rate (beats/min)	77 ±14	81, 12	0.808

**Table 2:** Echocardiographic data in diabetic and non-diabetic patients with chronic HF.

**Variable**	**Non-diabetic**	**Diabetic**	***P*** **value**
	**(n = 233)**	**(n =111)**	
Ejection fraction (%)	47 ±16	45 ±16	0.245
IVSd/BSA (cm)	0.6 ±0.1	0.6 ±0.1	0.388
Left atrium/BSA (cm)	2.4 ±0.5	2.4 ±0.4	0.952
LV EDD/BSA (cm)	3.2 ±0.8	3.1 ±0.7	0.135
LV ESD/BSA (cm)	2.4 ±0.7	2.4 ±0.6	0.740
Lateral MAPSE (cm)	1.2 ±0.4	1.2 ±0.3	0.955
Septal MAPSE (cm)	1.0 ±0.3	1.0 ±0.4	0.848
TAPSE (cm)	2.2 ±0.5	2.1 ±0.6	0.353
LV posterior wall/BSA (cm)	0.5 ±0.1	0.5 ±0.1	0.704
E wave (mm)	69 ±25	70 ±31	0.286
E/A ratio	1.2 ±0.8	1.2 ±0.9	0.804
Filling time (ms)	379 ±112	347 ±94	0.076
IVRT (ms)	103 ±27	117 ±36	0.068
E/e′ ratio	11.5 ±7.2	11 ±5.9	0.487
Lateral e′ (cm/s)	6.5 ±2.8	6.5 ±2.6	0.851
Lateral a′(cm/s)	7.5 ±3.2	7.6 ±3.1	0.876
Lateral s′ (cm/s)	5.6 ±1.9	5.7 ±1.7	0.088
Septal e′ (cm/s)	5.6 ±2.3	5.5 ±2.3	0.777
Septal a′ (cm/s)	7.5 ±2.5	7.7 ±3.6	0.586
Septal s′ (cm/s)	4.8 ±1.4	5.1 ±1.6	0.203
Right e′ (cm/s)	9.2 ±3.4	9.1 ±3.4	0.892
Right a′ (cm/s)	12.8 ±4.5	13.4 ±4.3	0.285
Right s′ (cm/s)	9.1 ±3.0	9.5 ±3.5	0.494

*LV, left ventricle; EDD, end-diastolic dimension; ESD, end-systolic dimension; IVSd, interventricular septum in diastole; MAPSE, mitral annular plane systolic excursion; TAPSE, tricuspid annular plane systolic excursion; A, atrial diastolic velocity; E, early diastolic filling velocity; e′, early diastolic myocardial velocity; s′, systolic myocardial velocity*.

### Patients With Good vs. Limited 6 MWT Performance

Patients with limited exercise capacity had a higher prevalence of T2DM (*p* < 0.001), arterial hypertension (*p* = 0.004), and atrial fibrillation (*p* = 0.001), a higher waist/hips ratio (*p* = 0.041), a level of fasting glucose (*p* < 0.001), and a lower level of hemoglobin (*p* < 0.001), compared with those with good exercise capacity. In addition, they had larger LA (*p* = 0.002), reduced lateral MAPSE (*p* = 0.032), septal MAPSE (*p* < 0.001), and TAPSE (*p* < 0.001), compared to patients with good 6-MWT performance. The rest of the clinical and echocardiographic indices were not different between subgroups (Tables 3, 4).

**Table 3:** Clinical and biochemical data in patients with limited exercise vs. good exercise capacity.

**Variable**	**6-MWT > 300 m**	**6-MWT ≤ 300 m**	***p*** **value**
	**(*n* = 168)**	**(*n* =176)**	
Age (years)	61 ±8	62 ±9	0.095
Female (%)	45	47	0.817
Smoking (%)	30	24	0.517
Diabetes (%)	21	43	<0.001
Arterial hypertension (%)	59	76	0.004
Atrial fibrillation (%)	10	24	0.001
Waist/hips ratio	0.96 ±0.08	0.96 ±0.08	0.041
Body-mass index (kg/m^2^)	28.6 ±4	28.2 ±5	0.069
Fasting glucose (mmol/L)	6.5 ±2.5	7.5 ±3.5	<0.001
Total cholesterol (mmol/L)	4.8 ±1.2	4.5 ±1.2	0.805
Triglycerides (mmol/L)	1.7 ±0.7	1.6 ±1.0	0.129
Creatinine (μmol/L)	99 ±50	99 ±31	0.975
Urea	8.5 ±4.5	9.3 ±4.5	0.147
Hemoglobin (g/dl)	12.9 ±1.6	12.1 ±1.8	<0.001
Baseline HR (beats/min)	81 ±15	76 ±12	0.808

*6-MWT, 6-min walk test*.

**Table 4:** Echocardiographic data in patients with limited exercise vs. good exercise capacity (6-MWT distance).

**Variable**	**6-MWT > 300 m**	**6-MWT ≤ 300 m**	***p*** **value**
	**(*n* = 168)**	**(*n* =176)**	
Ejection fraction (%)	47 ±17	45 ±16	0.245
IVSd/BSA (cm/m^2^)	0.6 ±0.1	0.6 ±0.1	0.407
Left atrium/BSA (cm/m^2^)	2.3 ±0.5	2.5 ±0.5	0.002
LV EDD/BSA (cm/m^2^)	3.1 ±0.6	3.2 ±0.6	0.065
LV ESD/BSA (cm/m^2^)	2.3 ±0.8	2.5 ±0.7	0.090
Lateral MAPSE (cm)	1.2 ±0.4	1.1 ±0.3	0.032
Septal MAPSE (cm)	1.1 ±0.3	0.9 ±0.4	<0.001
TAPSE (cm)	2.2 ±0.5	2.0 ±0.6	<0.001
LV posterior wall (cm/m^2^)	0.5 ±0.2	0.5 ±0.1	0.584
E wave (mm)	63 ±23	66 ±26	0.286
E/A ratio	1.2 ±0.8	1.2 ±0.9	0.804
DT of E wave (ms)	167 ±53	160 ±55	0.076
E/e′ ratio	10.8 ±5.4	11.9 ±8.0	0.487
Lateral e′ (cm/s)	6.5 ±2.6	6.4 ±2.8	0.851
Lateral a′(cm/s)	7.8 ±3.3	7.3 ±3.1	0.876
Lateral s′ (cm/s)	5.7 ±1.9	5.5 ±1.8	0.088
Septal e′ (cm/s)	5.5 ±2.1	5.5 ±2.4	0.777
Septal a′ (cm/s)	7.7 ±2.5	7.3 ±3.1	0.586
Septal s′ (cm/s)	4.8 ±1.4	4.8 ±1.6	0.203
Right e′ (cm/s)	9.1 ±3.4	9.0 ±3.7	0.892
Right a′ (cm/s)	12.7 ±4.0	13.3 ±4.8	0.285
Right s′ (cm/s)	9.2 ±2.8	9.3 ±3.5	0.494

*6-MWT, 6-min walk test; LV, left ventricle; EDD, end-diastolic dimension; ESD, end-systolic dimension; IVSd, interventricular septum in diastole; BSA, body-surface area; MAPSE, mitral annular plane systolic excursion; TAPSE, tricuspid annular plane systolic excursion; A, atrial diastolic velocity; E, early diastolic filling velocity; e′, early diastolic myocardial velocity; s′, systolic myocardial velocity; DT, deceleration time*.

### Predictors of Limited 6-MWT Distance in All Patients

In the univariate analysis model, T2DM (*p* < 0.001), low hemoglobin level (*p* < 0.001), atrial fibrillation (*p* < 0.001), and NYHA class (*p* = 0.008) predicted limited 6-MWT distance, as did enlarged LA (*p* = 0.003), increased E wave velocity (*p* = 0.019), raised E/e′ (*p* = 0.028), reduced lateral MAPSE (*p* = 0.033) septal MAPSE (*p* < 0.001), TAPSE (*p* < 0.001) and septal a′ and s′ (*p* = 0.032 and *p* = 0.041, respectively), and increased E/e′ (*p* = 0.028). In multivariate analysis [odds ratio (*OR*) 95% confidence interval (*CI*)], only diabetes [3.366 (1.907–5.939), *p* < 0.001], low hemoglobin [0.847 (0.729–0.985), *p* = 0.031], atrial fibrillation [2.684 (1.273–5.657), *p* = 0.009], and reduced septal MAPSE [0.308 (0.125–0.759), *p* = 0.010], independently predicted the limited 6-MWT distance (Table 5).

**Table 5:** Predictors of limited exercise in All patients with HF.

**Variable**	**OR**	**(CI 95%)**	***p*** **value**
**Univariate predictors**			
Age	1.021	(0.996–1.047)	0.098
Diabetes mellitus	2.723	(1.694–4.376)	< 0.001
NYHA class	1.472	(1.107–1.956)	0.008
Basal heart rate	0.973	(0.947–1.001)	0.055
Smoking	0.833	(0.517–1.344)	0.455
Gender	1.255	(0.801–1.874)	0.349
Left atrium/BSA	2.026	(1.274–3.220)	0.003
E wave	1.023	(1.004–1.043)	0.019
E/A	1.113	(0.862–1.437)	0.410
Hemoglobin	0.770	(0.675–0.878)	< 0.001
LV EDD/BSA	1.381	(0.979–1.950)	0.066
LV ESD/BSA	1.289	(0.961–1.370)	0.090
LV EF	0.995	(0.983–1.009)	0.496
Lateral MAPSE	0.500	(0.264–0.944)	0.033
Septal MAPSE	0.197	(0.088–0.439)	< 0.001
TAPSE	0.426	(0.276–0.657)	< 0.001
Lateral e′	0.995	(0.915–1.081)	0.900
Lateral a′	0.944	(0.875–1.081)	0.135
Lateral s′	0.940	(0.829–1.066)	0.332
Septal e′	1.009	(0.901–1.130)	0.874
Septal a′	0.949	(0.861–1.046)	0.289
Septal s′	1.007	(0.849–1.95)	0.934
BMI	0.981	(0.937–1.027)	0.406
Atrial fibrillation	2.784	(1.513–5.121)	0.001
Arterial hypertension	0.877	(0.563–1.365)	0.561
Creatinine	1.000	(0.994–1.006)	0.975
E/e′	1.092	(1.009–1.181)	0.028
Septal a′	0.786	(0.631–0.979)	0.032
Septal s′	0.661	(0.444–0.984)	0.041
**Multivariate predictors**			
Type 2 diabetes mellitus	3.366	(1.907–5.939)	< 0.001
Hemoglobina	0.847	(0.729–0.985)	0.031
Atrial fibrillation	2.684	(1.273–5.657)	0.009
Septal MAPSE	0.308	(0.125–0.759)	0.010
TAPSE	0.998	(0.598–1.679)	0.994
Left atrium diameter/BSA	1.771	(0.970–3.108)	0.064
NYHA class	1.167	(0.821–1.658)	0.389

*LV, left ventricle; EDD, end-diastolic dimension; ESD, end-systolic dimension; MAPSE, mitral annular plane systolic excursion; TAPSE, tricuspid annular plane systolic excursion; A, atrial diastolic velocity; E, early diastolic filling velocity; e′, early diastolic myocardial velocity; s′, systolic myocardial velocity; DT, deceleration time; BSA, body-surface area; NYHA, New York Heart Association*.

### Predictors of Limited 6 MWT Distance in HFpEF

Type 2 diabetes mellitus (*p* < 0.001), low hemoglobin (*p* = 0.022), atrial fibrillation (*p* = 0.020), NYHA class (*p* = 0.005), LA (*p* = 0.03), septal MAPSE (*p* < 0.001), and lateral MAPSE (*p* = 0.044) predicted limited 6-MWT distance in HFpEF. In multivariate analysis [*OR* 95% *CI*], only diabetes [6.083 (2.613–14.160), *p* < 0.001], atrial fibrillation [6.092 (1.769–20.979), *p* = 0.004], and septal MAPSE [0.063 (0.027–0.184), *p* = 0.002], independently predicted the limited 6-MWT distance in HFpEF (Tables 6, 7).

**Table 6:** Univariate predictors of limited exercise capacity (6-MWT < 300 m) in patients with non-reduced and those with reduced left ventricular ejection fraction (LVEF).

**Variable**	**HF Patients with**			**HF Patients with**		
	**LVEF ≥ 40%**			**LVEF < 40%**		
	**OR**	**(CI 95%)**	***p value***	**OR**	**(CI 95%)**	***p value***
Age	1.027	(0.989–1.066)	0.171	1.017	(0.983–1.051)	0.327
Gender	1.158	(0.627–2.138)	0.639	1.391	(0.747–2.593)	0.299
Basal heart rate	0.974	(0.915–1.036)	0.397	0.976	(0.946–1.007)	0.132
Duhani	0.865	(0.410–1.826)	0.703	0.772	(0.407–1.467)	0.430
Arterial hypertension	0.796	(0.397–1.554)	0.488	0.990	(0.536–1.830)	0.976
BMI	0.978	(0.925–1.033)	0.423	0.989	(0.910–1.074)	0.784
BSA	0.156	(0.024–1.017)	0.052	0.421	(0.071–2.516)	0.343
NYHA class	1.892	(1.202–2.783)	0.005	1.207	(0.779–1.870)	0.400
Atrial fibrillation	3.727	(1.527–8.837)	0.020	2.029	(0.852–4.832)	0.110
Diabetes	3.929	(2.009–7.682)	<0.001	1.840	(0.935–3.622)	0.078
Creatinine	1.011	(0.997–1.026)	0.128	0.997	(0.990–1.004)	0.411
Hemoglobin	0.811	(0.679–0.970)	0.022	0.726	(0.599–0.882)	0.001
Left atrium dimension/BSA	2.041	(1.070–3.893)	0.030	2.127	(1.025–4.415)	0.043
LV EDD/BSA	1.719	(0.835–3.538)	0.142	1.434	(0.832–2.471)	0.195
LV ESD/BSA	1.823	(0.863–3.851)	0.116	1.466	(0.841–2.553)	0.177
LV EF	0.986	(0.957–1.016)	0.350	1.008	(0.966–1.051)	0.722
E wave	1.008	(0.995–1.021)	0.249	1.004	(0.992–1.016)	0.508
E/A ratio	0.926	(0.547–1.566)	0.774	1.166	(0.849–1.601)	0.343
Lateral MAPSE	0.376	(0.145–0.975)	0.044	0.772	(0.301–1.980)	0.591
Septal MAPSE	0.092	(0.027–0.314)	<0.001	0.432	(0.128–1.464)	0.178
TAPSE	0.578	(0.306–1.091)	0.091	0.309	(0.157–0.610)	0.001
Lateral e′	0.972	(0.865–1.092)	0.627	1.069	(0.941–1.214)	0.304
Lateral a′	0.897	(0.802–1.003)	0.058	1.013	(0.907–1.130)	0.824
Lateral s′	0.991	(0.809–1.214)	0.933	0.929	(0.791–1.092)	0.374
Septal e′	1.018	(0.887–1.168)	0.801	1.097	(0.874–1.377)	0.426
Septal a′	0.984	(0.874–1.109)	0.792	0.924	(0.780–1.093)	0.356
Septal s′	1.049	(0.823–1.338)	0.697	1.031	(0.797–1.332)	0.818
E/e′	1.027	(0.974–1.083)	0.990	1.019	(0.972–1.068)	0.430

*BMI, body-mass index; BSA, body-surface area; LV, left ventricle; EDD, end-diastolic dimension; ESD, end-systolic dimension; IVSd, interventricular septum in diastole; MAPSE, mitral annular plane systolic excursion; TAPSE, tricuspid annular plane systolic excursion; A, atrial diastolic velocity; E, early diastolic filling velocity; e′, early diastolic myocardial velocity; s′, systolic myocardial velocity*.

**Table 7:** Multivariate predictors of limited exercise capacity (6-MWT <300 m) in patients with non-reduced and those with reduced LVEF.

**Variable**	**HF patients with**			**HF patients with**		
	**LVEF ≥40%**			**LVEF < 40%**		
	**OR**	**(CI 95%)**	***p value***	**OR**	**(CI 95%)**	***p value***
Diabetes	6.083	(2.613–14.160)	<0.001	1.587	(0.656–3.732)	0.342
Hemoglobin	0.870	(0.693–1.092)	0.230	0.786	(0.624–0.998)	0.049
Atrial fibrillation	6.092	(1.769–20.979)	0.004	1.312	(0.474–3.594)	0.622
Septal MAPSE	0.063	(0.027–0.184)	0.002	0.892	(0.218–3.497)	0.812
TAPSE	0.779	(0.344–1.766)	0.550	0.462	(0.214–0.988)	0.041
Left atrium diameter/BSA	1.198	(0.498–2.883)	0.687	1.352	(0.854–2.116)	0.246
NYHA class	1.034	(0.597–1.791)	0.905	1.184	(0.745–1.998)	0.592

*6-MWT, 6-min walk test; MAPSE, mitral annular plane systolic excursion; TAPSE, tricuspid annular plane systolic excursion; BMI, body-mass index; BSA, body-surface area; NYHA, New York Heart Association*.

### Predictors of Limited 6 MWT Distance in Patients With HFrEF

In univariate analysis, low hemoglobin (*p* = 0.001), reduced TAPSE (*p* = 0.001), and enlarged LA (*p* = 0.043) predicted limited 6-MWT distance in patients with HFrEF. In multivariate analysis, only low hemoglobin [0.786 (0.624–0.998), *p* = 0.049] and reduced TAPSE [0.462 (0.214–0.988), *p* = 0.041] independently predicted the limited 6-MWT distance in HFrEF (Tables 6, 7).

## Discussion

### Findings

Heart failure patients with limited exercise capacity had a higher prevalence of T2DM, arterial hypertension, and atrial fibrillation, compared with those with good exercise capacity. They also had a higher waist/hips ratio, lower hemoglobin, a larger LA, and compromised LV and RV long-axis systolic function. Patients with combined HF and T2DM had a higher prevalence of arterial hypertension, a higher waist/hips ratio, more compromised renal function, lower hemoglobin, and a shorter 6-MWT distance, compared with those with HF with no T2DM. Multivariate analysis showed diabetes, lowered hemoglobin level, atrial fibrillation, and reduced LV long-axis function as independent predictors of limited exercise capacity in patients with HF.

While the above common knowledge on the relationship between atherosclerosis risk factors and HF is confirmed in our patients, their impact on predicting exercise capacity differed significantly according to LVEF. In patients with reduced LVEF, low hemoglobin and compromised RV long-axis systolic function were the two independent predictors of exercise capacity. However, in patients with LV preserved EF, T2DM, low hemoglobin level, atrial fibrillation, higher NYHA class, and compromised LV long-axis systolic function were the respective predictors.

### Data Interpretation

Exercise intolerance is the main symptom in patients with HF, regardless of LVEF (32, 33). In these patients, different echocardiographic indices have been shown as important predictors of exercise capacity, particularly raised LV filling pressures (34–42). Such a relationship can be explained on the basis of reduced stroke volume and pulmonary venous hypertension (43). This is, however, only one explanation of exercise intolerance in HF. Our results provide a clearer image as to the potential mechanisms involved in reduced exercise capacity in patients with HF, when they are classified according to EF. HF with reduced EF is commonly caused by ischemic myopathy that involves both ventricles with their impact on cardiac output and kidney function (44). Indeed, our findings confirm that, having shown that low hemoglobin (45) and compromised RV systolic function (46) are the two main predictors of limited exercise. However, in patients with preserved LVEF, the scenario differs having shown that T2DM and atrial fibrillation are two additional predictors of exercise capacity. Atrial fibrillation is a very common finding in HFpEF. Most such patients are known to have long-standing hypertensive LV disease and left atrial enlargement with its known complications (47). Atrial fibrillation loses the atrial systolic filling component of the LV, hence compromising overall stroke volume with its impact on exercise capacity (48). T2DM, on the other hand, enhances the atherosclerosis pathology at both main coronary arteries level as well as microcirculation with resulting subendocardial fibrosis and LV cavity stiffness, raising the filling pressures and eventually causing atrial fibrillation (49). Through the same atherosclerotic pathophysiology, T2DM also impacts the peripheral circulation and, over the years, causes peripheral neuropathy. Although our analysis identified individual independent predictors of the limited exercise capacity in patients with HF, it must be mentioned that the pathomechanisms are closely related, particularly T2DM and atrial fibrillation, and their impact on LV myocardial function with its consequence on left atrial enlargement, atrial fibrillation, and its complications.

### Limitations

The main limitation of our study is that we did not assess the response of echocardiographic measurements to exercise, at the time of symptoms development. However, the main objective of the study was to identify predictors of ordinary walking exercise limitations rather than a heavy exercise in patients with HF. The lack of left atrial pressure invasive measurements is another limitation, but the study was based on conventional Doppler measurements, which have been shown to be reproducible and correlate closely with invasive pressure measurements (50). We did not have myocardial deformation measurements in our cohort, which might have altered the results.

### Clinical Implications

Type 2 diabetes mellitus has a significant impact on exercise intolerance in patients with HFpEFEF. While the cardiac pump function looks better preserved than in patients with HFrEF, the multi-system complications associated with diabetes should be acknowledged, particularly myocardial microcirculation and peripheral arterial disease as well as peripheral neuropathic complications.

## Conclusion

Predictors of exercise intolerance in patients with chronic HF differ according to LV systolic function, judged as EF. T2DM seems the most powerful predictor of limited exercise capacity in patients with HFpEF.

## Data Availability Statement

The original contributions presented in the study are included in the article/supplementary material, further inquiries can be directed to the corresponding author/s.

## Ethics Statement

The studies involving human participants were reviewed and approved by Ethical Committee, Medical Faculty, University of Prishtina, Prishtina, Kosovo. The patients/participants provided their written informed consent to participate in this study.

## Author Contributions

VB-H, MH, SE, and GB contributed to conception and design of the study. IB, PI, EH, AP, RT, and AB organized the database. GB, PI, and IB performed the statistical analysis. VB-M wrote the first draft of the manuscript. GB, IB, MH, and SE wrote sections of the manuscript. All authors contributed to manuscript revision, read, and approved the submitted version.

## Conflict of Interest

The authors declare that the research was conducted in the absence of any commercial or financial relationships that could be construed as a potential conflict of interest.

## Publisher's Note

All claims expressed in this article are solely those of the authors and do not necessarily represent those of their affiliated organizations, or those of the publisher, the editors and the reviewers. Any product that may be evaluated in this article, or claim that may be made by its manufacturer, is not guaranteed or endorsed by the publisher.
